# Decursin Suppresses Esophageal Squamous Cell Carcinoma Progression via Orchestrated Cell Cycle Deceleration, Apoptotic Activation, and Oncoprotein Degradation

**DOI:** 10.3390/ijms26115391

**Published:** 2025-06-04

**Authors:** Chen Fang, Lin Wu, Xiangzhe Yang, Kai Xie, Peng Zhang, Yu Feng, Haitao Ma, Xing Tong

**Affiliations:** 1 Department of Thoracic and Cardiovascular Surgery, Medical Center of Soochow University, Suzhou 215000, China; fangchensuda@163.com (C.F.); lin_wu50@163.com (L.W.); 20234256009@stu.suda.edu.cn (X.Y.); kaixie715786420@126.com (K.X.); 2 Advanced Molecular Pathology Institute of Soochow University and SANO, Suzhou 215128, China; pzhang1995@sanopm.com; 3 Department of Thoracic Surgery, The First Clinical Medical College of Soochow University, Suzhou 215006, China; fengyu1@suda.edu.cn; 4 Department of Pathology, the First Affiliated Hospital of Soochow University, Suzhou 215006, China

**Keywords:** decursin, ESCC, natural compound, cancer therapy

## Abstract

Esophageal squamous cell carcinoma (ESCC) remains a lethal malignancy with limited therapeutic options. This study investigated the antitumor efficacy and mechanisms of decursin, a natural pyranocoumarin derivative, against ESCC. In vitro analyses demonstrated that decursin selectively inhibited ESCC cell viability (IC50: 14.62 ± 0.61–26.20 ± 2.11 μM across TE-1, KYSE-30, and KYSE-150 cell lines) without affecting normal esophageal epithelial cells (Het-1A). Decursin (10 μM) suppressed colony formation, impaired wound healing (*p* < 0.001 at 48 h), and reduced Transwell migration/invasion in KYSE-150 cells. Subcutaneous xenograft models revealed significant tumor growth inhibition (*p* < 0.01) with decursin treatment (10 mg/kg, intraperitoneal), accompanied by no systemic toxicity. Mechanistically, decursin induced G0/G1 cell cycle deceleration (*p* < 0.01) and apoptosis through ubiquitin–proteasome-mediated degradation of oncoproteins TP63 and SOX2. Time- and dose-dependent protein suppression was reversed by proteasome inhibitor MG-132, but unaffected by lysosomal inhibition. These findings establish decursin as a promising therapeutic agent for ESCC, functioning via proteasomal degradation of key oncogenic drivers, and provide a rationale for decursin’s further development as a targeted monotherapy or chemosensitizer in multimodal regimens.

## 1. Introduction

Esophageal cancer represents a highly aggressive thoracic malignancy, ranking as the seventh leading cause of cancer-related mortality worldwide [1,2]. Its primary pathological subtypes include esophageal squamous cell carcinoma (ESCC) and esophageal adenocarcinoma (EAC), with ESCC accounting for approximately 90% of cases [3]. Current therapeutic strategies primarily involve surgery-based multimodal approaches incorporating chemotherapy, radiotherapy, immunotherapy, and targeted therapies [4,5]. Despite recent advancements in treatment modalities, the prognosis remains dismal, with a 5-year survival rate of merely 22%, underscoring the urgent need for novel therapeutic interventions to improve clinical outcomes [1,6].

**Figure 1:**
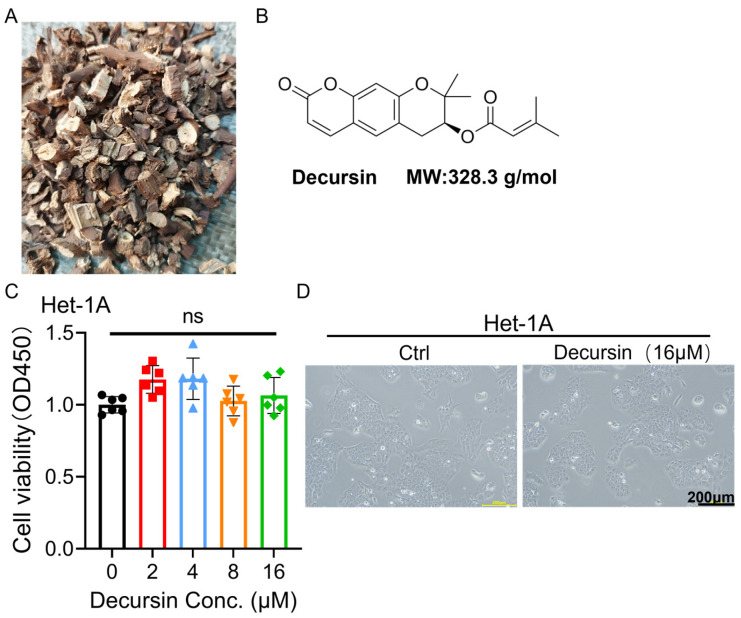
(**A**) Angelica gigas (Umbelliferae) roots. (**B**) Decursin chemical structure. (**C**) Cell viability of normal esophageal epithelial cells (HET-1A) at different concentrations of decursin. (**D**) Cell morphology of normal esophageal epithelial cells (HET-1A) before and after decursin treatment. MW: molecular weight; ns: no significance.

Natural compounds, particularly those derived from traditional Chinese medicine, have demonstrated substantial antitumor potential across various malignancies. Notable examples include curcumin and emodin in breast cancer suppression [7,8], emodin and esculin in colorectal carcinoma management [9,10], and Triptolide’s cytotoxic effects against gastric cancer cells [11]. Decursin, a bioactive coumarin derivative isolated from Angelica gigas (Umbelliferae) roots (Figure 1A), exhibits multifaceted pharmacological properties encompassing anticancer, anti-angiogenic, and anti-inflammatory activities [12,13]. Previous investigations by Yun Ge et al. revealed that decursin inhibits tumor angiogenesis through hypoxia-inducible factor 1α (HIF-1α) ubiquitination-mediated degradation, subsequently suppressing vascular endothelial growth factor (VEGF) and carbonic anhydrase IX (CA9) expression. Concurrently, it enhances CD8+ T cell infiltration within the tumor microenvironment while inhibiting PD-L1 expression and immunosuppressive cell populations such as regulatory T cells [14]. Additional mechanistic studies demonstrated decursin’s capacity to suppress gastric cancer progression via CXCR7/STAT3/c-Myc signaling pathway inhibition and caspase-3-dependent apoptosis induction [15]. Despite these promising antitumor properties, the therapeutic potential of decursin in esophageal carcinoma remains unexplored.

This study systematically investigated the biological effects of decursin on ESCC progression and conducted preliminary mechanistic exploration, aiming to address this critical knowledge gap in esophageal cancer research.

## 2. Results

### 2.1. Decursin Spares Normal Esophageal Epithelium While Suppressing ESCC Viability

**Figure 2:**
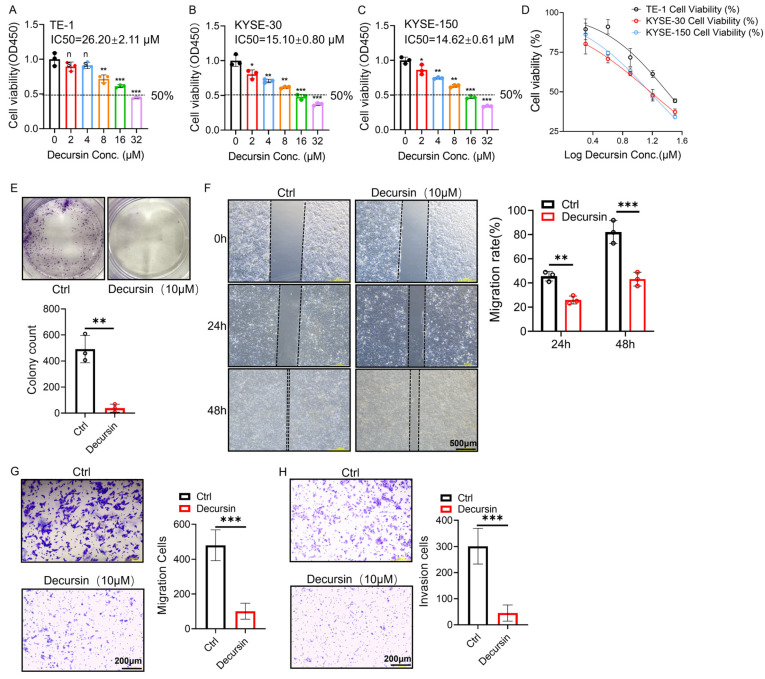
(**A**–**D**) Cell viability and IC50 of ESCC cell lines TE-1, KYSE-30, and KYSE-150 at different decursin concentrations. (**E**) Colony formation assay to detect the effect of decursin on the proliferation of ESCC cells. (**F**,**G**) Wound healing and Transwell assays to detect the effect of decursin on the migration of ESCC cells. (**H**) Transwell assay to detect the effect of decursin on the invasion of ESCC cells. IC50: half-maximal inhibitory concentration; ns: no significance; *, *p* < 0.05; **, *p* < 0.01; ***, *p* < 0.001.

The CCK-8 assay revealed that decursin exhibited no significant impact on the viability of normal esophageal epithelial cells (Het-1A) at concentrations ranging from 0 to 16 μM (Figure 1C), with preserved cellular morphology confirmed by microscopic observation (Figure 1D). In contrast, dose-dependent inhibition of cell viability was observed in all three esophageal squamous cell carcinoma (ESCC) lines (TE-1, KYSE-30, and KYSE-150), with calculated IC50 values of 26.20 ± 2.11 μM, 15.10 ± 0.80 μM, and 14.62 ± 0.61 μM, respectively (Figure 2A–D). Based on these findings, KYSE-150 cells—the most sensitive to decursin—were selected for subsequent experiments using a concentration of 10 μM, approximately equivalent to its IC50 value.

### 2.2. Decursin Suppresses Proliferation, Migration, and Invasion in ESCC Cells

Colony formation assays demonstrated marked suppression of KYSE-150 cell clonogenicity in the decursin-treated group (10 μM) compared to controls (Figure 2E, *p* < 0.01), indicating impaired proliferative capacity. Wound healing analysis revealed time-dependent inhibition of migration, with significant reduction in scratch closure rates observed at 24 h (*p* < 0.01) and more pronounced effects at 48 h post-treatment (Figure 2F, *p* < 0.001). Consistent with these findings, Transwell migration assays confirmed attenuated cell motility under decursin exposure (Figure 2G, *p* < 0.001). Furthermore, Matrigel-based invasion assays substantiated decursin’s inhibitory effects on ESCC cell invasiveness (Figure 2H, *p* < 0.001).

### 2.3. Decursin Attenuates ESCC Progression In Vivo

**Figure 3:**
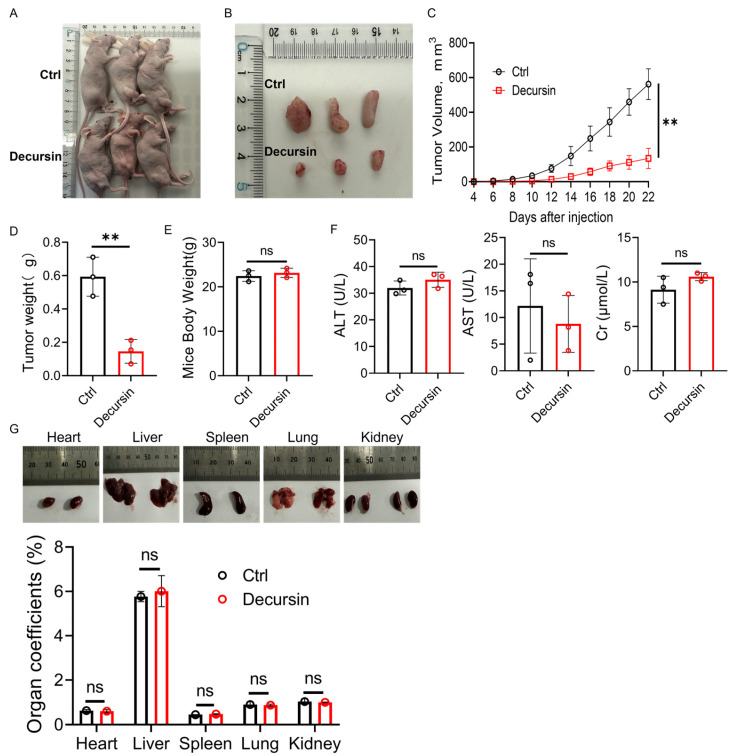
(**A**) Tumor-bearing nude mice. (**B**) Appearance of subcutaneous tumors. (**C**) Volume of subcutaneous tumors in nude mice. (**D**) Weight of subcutaneous tumors in nude mice. (**E**) Weight of nude mice at the end of the experiment. (**F**) Mice serum biochemical analysis (ALT, AST, and Cr). (**G**) Appearance of organs and organ coefficients in nude mice. ns: no significance; **, *p* < 0.01.

To validate the anti-tumor efficacy of decursin in vivo, we established subcutaneous xenograft models by inoculating KYSE-150 cells into the right leg of nude mice (Figure 3A). Tumor growth analysis revealed that the decursin-treated group exhibited significantly attenuated tumor progression compared to controls (Figure 3C, *p* < 0.01). At the study’s termination, marked reductions in both tumor volume and weight were observed in the decursin cohort (Figure 3B,D). Computational toxicity prediction using the ProTox-II platform (https://tox-new.charite.de/, accessed on 20 May 2025) indicated the low systemic toxicity of decursin (S1). Serum biochemical analysis further demonstrated that decursin treatment did not significantly alter levels of alanine aminotransferase (ALT), aspartate aminotransferase (AST), or creatinine (Cr) compared to the control group (Figure 3F). Notably, no significant differences in body weight or organ-to-body weight ratios (heart, liver, spleen, lungs, and kidneys) were detected between groups (Figure 3E,G), confirming both in silico predictions and experimental observations of decursin’s favorable safety profile.

### 2.4. Decursin Suppresses ESCC via Dual Mechanisms: Cell Cycle Deceleration and Apoptosis Induction

**Figure 4:**
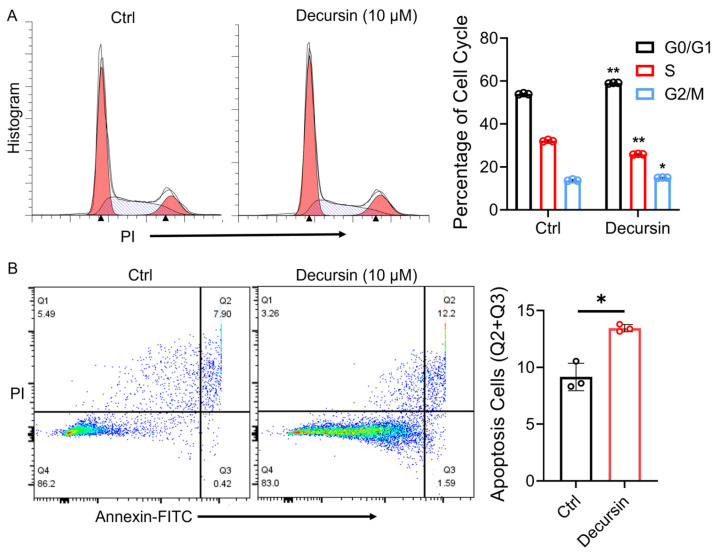
(**A**) Flow cytometry was used to detect cell cycle changes in esophageal squamous cell carcinoma cells before and after decursin treatment. (**B**) Flow cytometry was used to detect apoptosis of esophageal squamous cell carcinoma cells before and after decursin treatment. *, *p* < 0.05; **, *p* < 0.01.

Cell cycle analysis demonstrated decursin-induced G0/G1 phase deceleration in ESCC cells (Figure 4A, *p* < 0.01), indicating proliferation suppression through cell cycle modulation. In parallel, apoptosis assays revealed significant induction of programmed cell death following decursin treatment (Figure 4B, *p* < 0.01).

### 2.5. Decursin Promotes Oncoprotein Degradation Through Dose–Time Dependency and Ubiquitin–Proteasome Activation

**Figure 5:**
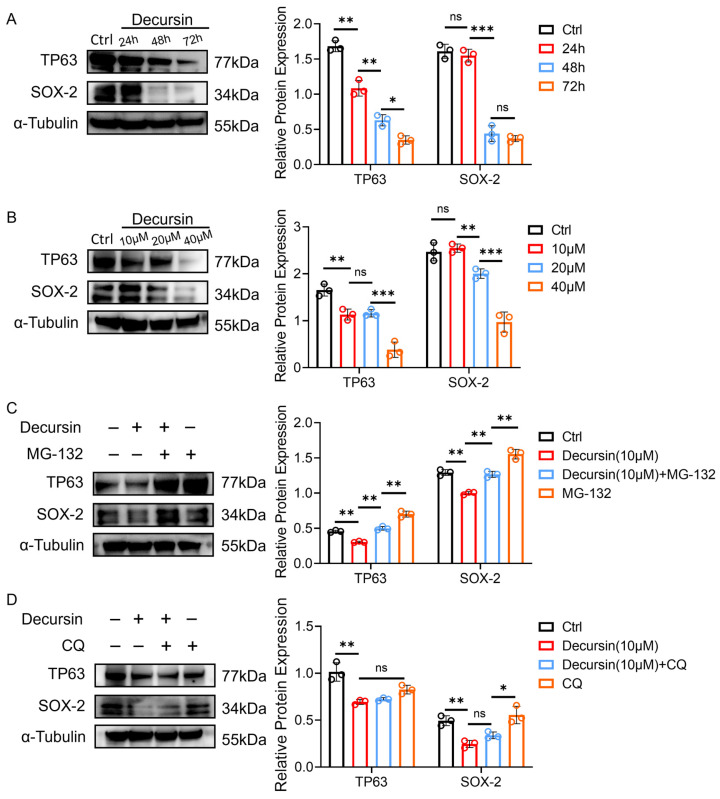
(**A**) Time-dependent effects of decursin exposure on ESCC-related malignant protein expression (concentration maintained at 10 μM). (**B**) Concentration-dependent effects of decursin on ESCC-related malignant protein expression (treatment duration fixed at 24 h). (**C**) Effects of the combined use of proteasome inhibitor MG-132 and decursin on the expression of ESCC-related malignant proteins. (**D**) Effects of the combined use of lysosome inhibitor chloroquine (CQ) on the expression of ESCC-related malignant proteins. ns: no significance; *, *p* < 0.05; **, *p* < 0.01; ***, *p* < 0.001.

To investigate the tumor-suppressive mechanisms of decursin, we performed Western blot analysis of ESCC-associated oncoproteins TP63 and SOX-2 [16,17]. Time-course experiments (with decursin concentration maintained at 10 μM) demonstrated progressive downregulation of both proteins at 24, 48, and 72 h post-treatment (Figure 5A, *p* < 0.01), indicating time-dependent therapeutic effects. Concurrently, dose–response analysis (with treatment duration fixed at 24 h) revealed concentration-dependent reduction in TP63/SOX-2 expression levels at 10, 20, and 40 μM of decursin (Figure 5B, *p* < 0.01).

To elucidate the degradation pathway, we co-administered decursin with proteasome inhibitor MG-132 or lysosome inhibitor chloroquine (CQ). MG-132 pretreatment substantially reversed decursin-induced TP63/SOX-2 suppression (Figure 5C), whereas CQ showed no inhibitory effect on protein downregulation (Figure 5D). These findings substantiate that decursin promotes ubiquitin–proteasome system-mediated degradation of ESCC oncoproteins, rather than through lysosomal pathways.

## 3. Discussion

In this study, we experimentally demonstrated the therapeutic potential of the natural product decursin in esophageal carcinoma and conducted a preliminary exploration of its mechanism of action. Results revealed that decursin significantly inhibited the proliferation, migration, and invasion of esophageal squamous cell carcinoma (ESCC), with determined IC50 values of 26.20 ± 2.11 μM, 15.10 ± 0.80 μM, and 14.62 ± 0.61 μM for TE-1, KYSE-30, and KYSE-150 cell lines, respectively. The tumor-suppressive efficacy of decursin was further validated in a nude mouse subcutaneous xenograft model. Notably, at tumor-inhibitory concentrations, decursin exhibited no discernible adverse effects on normal cells and organ tissues, indicating its favorable safety profile. Preliminary mechanistic investigations demonstrated that decursin treatment induced significant G0/G1 phase cell cycle deceleration in ESCC cells, accompanied by enhanced apoptosis as confirmed by flow cytometry analysis. To further elucidate the molecular basis underlying decursin’s anti-tumor effects, we examined two malignancy-associated proteins (TP63 and SOX-2) under varying treatment durations (0 h, 24 h, 48 h, and 72 h) and concentrations (0 μM, 10 μM, 20 μM, and 40 μM). Both proteins exhibited progressive degradation in a time- and dose-dependent manner following decursin administration. Subsequent combination experiments with proteasome inhibitor MG-132 and lysosome inhibitor chloroquine demonstrated that MG-132 effectively reversed decursin-mediated oncoprotein degradation, whereas chloroquine showed no such effect. These findings collectively suggest that decursin exerts its anti-cancer effects primarily through ubiquitin–proteasome pathway activation rather than lysosomal degradation mechanisms.

Esophageal carcinoma, particularly its predominant subtype esophageal squamous cell carcinoma (ESCC), has induced a significant global health challenge and economic burden due to its rapid progression and limited therapeutic options [18,19]. Conventional treatments such as radiotherapy and chemotherapy are frequently associated with substantial side effects, often resulting in suboptimal prognosis and five-year survival rates [20,21]. These limitations underscore the critical need for developing novel therapeutic agents with enhanced efficacy and reduced toxicity in oncology research [22]. Notably, plant-derived natural products have been integral to traditional Chinese medicine for millennia [23], with modern technological advancements enabling the identification of bioactive compounds from herbal sources for anticancer applications [23,24]. Decursin, a natural coumarin derivative isolated from Angelica gigas (Umbelliferae) roots, has demonstrated promising antitumor properties in gastric and colorectal cancers [25,26,27]. However, its therapeutic potential in ESCC—another major malignancy of the digestive system—had remained unexplained. Our study not only addresses this knowledge gap, but also provides mechanistic insights into decursin’s anticancer activity.

The molecular pathogenesis of ESCC involves dysregulation of key oncogenic drivers, particularly TP63 and SOX2, which coordinate multiple facets of squamous carcinogenesis [16]. TP63, functioning as a central regulator of squamous epithelial differentiation, promotes tumor progression through dual mechanisms—activating proliferative pathways (NOTCH and Wnt/β-catenin signaling) while inhibiting apoptosis mediators like BAX and PUMA [16,28,29]. Clinically, elevated TP63 expression correlates with advanced disease stage and metastatic spread, establishing its value as both a diagnostic marker and prognostic predictor. Complementing this pathway, the stemness factor SOX2 maintains cancer cell plasticity and therapy resistance through regulation of stem cell markers including CD44 and OCT4 [16,30]. Our mechanistic investigations revealed that decursin targets both oncoproteins through a previously unrecognized mechanism: time- and dose-dependent induction of proteasomal degradation. This discovery extends decursin’s documented anticancer mechanisms beyond colorectal cancer (ROS/ER stress-mediated apoptosis) [27], gastric cancer (CXCR7-STAT3-cMyc axis suppression) [15], and hypoxic lung tumors (HIF-1α destabilization) [14], establishing proteasome-mediated oncoprotein degradation as a novel therapeutic modality in ESCC. Notably, the compound’s selective cytotoxicity toward malignant cells while sparing normal esophageal epithelium mirrors its favorable safety profile observed in gastrointestinal models [15,27], significantly enhancing its clinical translation potential.

Beyond monotherapeutic applications, plant-derived compounds have emerged as potential chemosensitizers to enhance conventional anticancer therapies [31,32]. For instance, the combination of resveratrol, quercetin, and catechin with gefitinib synergistically improved antitumor and antimetastatic effects in xenograft models [33]. Similarly, La et al. demonstrated that 50 μM of epigallocatechin gallate (EGCG) sensitized DLD1 colorectal cancer cells to 5-fluorouracil by modulating GRP78, NF-κB, miR-155-5p, and MDR1 pathways [34]. In cervical cancer, quercetin potentiated cisplatin-induced apoptosis through coordinated downregulation of MMP2, METTL3, P-Glycoprotein (P-Gp), and ezrin [35]. These findings collectively highlight the multifaceted mechanisms through which phytochemicals can enhance chemotherapeutic efficacy while mitigating adverse effects. Building on this scientific foundation, future investigations will explore decursin’s potential as a chemosensitizer for first-line esophageal cancer therapeutics. This strategy could enable dose reduction of conventional agents, achieving the dual objectives of enhanced therapeutic efficacy and reduced treatment-related toxicity.

In summary, this study confirms the antitumor efficacy of decursin against esophageal squamous cell carcinoma. By activating the ubiquitin–proteasome pathway, decursin promotes degradation of key oncoproteins such as TP63 and SOX-2, thereby inducing G0/G1 phase cell cycle deceleration and apoptosis. These molecular events collectively inhibit critical tumorigenic behaviors, including the proliferation, migration, and invasion of cancer cells.

## 4. Materials and Methods

### 4.1. Cell Lines and Reagents

The human normal esophageal epithelial cell line Het-1A and three esophageal squamous carcinoma cell lines (TE-1, KYSE-30, and KYSE-150) were obtained from EallBio Technologies (Beijing, China). Decursin (purity > 98%) was procured from Nanjing Bencao Yikang Biotech Co., Ltd. (Nanjing, China), with proteasome inhibitor MG-132 and autophagy inhibitor chloroquine both purchased from MedChemExpress (MCE, Monmouth Junction, NJ, USA). Cell Counting Kit-8 (CCK-8) and 0.5% crystal violet staining solution were sourced from Absin Bioscience Inc. (Shanghai, China) and Yisheng Biotech (Shanghai, China), respectively. Transwell migration assays were conducted using Corning chambers (New York, NY, USA), with apoptosis analysis performed via the Annexin V-FITC/PI Apoptosis Detection Kit (BD Biosciences, New York, NY, USA) and cell cycle progression assessed using Lianke Biotech’s detection kit (Hangzhou, China). Western blotting reagents, including precast gels and transfer membranes, were acquired from Epizyme Biopharmaceutical Technology (Shanghai, China), with antibodies against TP63 (cat# 12143-1-AP), SOX2 (cat# 20118-1-AP), and α-tubulin (cat# 11224-1-AP) purchased from the Proteintech Group (Wuhan, China).

### 4.2. Animal Experiment

Six BALB/c nude mice were obtained from Hangzhou Ziyuan Experimental Animal Technology Co., Ltd. (Hangzhou, China). All experimental protocols were approved by the Medical Ethics Committee of the Fourth Affiliated Hospital of Soochow University (Ethics No. 241136). Subcutaneous tumor models were established by injecting 1.5 × 10^6^ tumor cells in 50 μL suspension into each mouse’s right hind leg. Mice received intraperitoneal injections of 100 μL decursin solution (10 mg/kg) on days 1, 3, 5, and 7 post-implantation. Tumor volumes were monitored using the formula (L × W^2^)/2, where L and W represent the longest and shortest diameters, respectively. When tumors reached approximately 10 mm in diameter, all mice were euthanized for tumor and organ collection. Peritoneal blood samples were sent to the Hematology Center, Cyrus Tang Medical Institute, for serum biochemical analysis (including ALT, AST, and Cr) to evaluate the biotoxicity of decursin.

### 4.3. CCK-8 Assay

Cells in logarithmic phase were trypsinized and resuspended in complete medium (5 × 10^4^ cells/mL); 100 μL cell suspension (5 × 10^3^ cells/well) was plated in 96-well plates. After 24 h of incubation (37 °C, 5% CO_2_), adherent cells were treated with decursin at indicated concentrations for 24 h. Next, 10 μL CCK-8 solution was added, followed by 1–4 h of incubation. Absorbance at 450 nm was measured using a spectrophotometer. Six replicates were analyzed per treatment.

### 4.4. Colony Formation

Logarithmic phase cells were resuspended at 5 × 10^3^ cells/well in 2 mL complete medium and seeded into 6-well plates. Experimental groups received 10 μM of decursin supplementation during the 14-day culture period with regular medium replacement. After 14 days, cells were washed with PBS, fixed with 4% paraformaldehyde (15 min), and stained with 0.1% crystal violet (10 min). Following PBS rinsing to remove residual dye, colonies were photographed and quantified. Three independent replicates were analyzed per sample.

### 4.5. Wound Healing

Logarithmic phase cells were seeded in 6-well plates at 5 × 10^5^ cells/well in 2 mL of culture medium. After 24 h of incubation for adhesion, scratches were created using sterile pipette tips. Experimental groups received 10 μM of decursin. Wound closure was monitored by microscopy at 0, 24, and 48 h. Three independent replicates were analyzed per condition.

### 4.6. Transwell Assay

Cells in logarithmic growth phase underwent serum starvation using serum-free medium for 24 h. For migration analysis, 500 μL of complete medium containing 10% fetal bovine serum was added to the lower chambers of Transwell inserts (8 μm pore), while upper chambers received 200 μL of serum-free medium containing 1 × 10^5^ cells/mL. Invasion assays incorporated an additional preliminary step in which upper chamber membranes were coated with 100 μL of Matrigel matrix (1:8 dilution in serum-free medium) and polymerized at 37 °C for 2 h prior to cell seeding. Both assay types proceeded with 24–48 h of incubation under standard culture conditions (37 °C, 5% CO_2_). Chambers were subsequently rinsed with PBS, fixed in 4% paraformaldehyde (10 min), and stained with 0.1% crystal violet (10 min). Following thorough PBS washing, transmigrated cells were quantified microscopically. Three experimental replicates were performed per sample.

### 4.7. Cell Apoptosis Profiling

Adherent cells were treated with 10 μL of decursin (10 μM) in experimental groups or an equivalent DMSO in controls. After 24 h of incubation, both supernatant and trypsinized adherent cells were collected from each sample. Following centrifugation and two cold PBS washes, cells were resuspended at 1 × 10^6^ cells/mL. Aliquots (100 μL) were transferred to flow cytometry tubes and stained with 5 μL of Annexin V-FITC and 5 μL of PI. Single-stain controls were prepared for compensation adjustments. After 15 min of dark incubation at room temperature, 400 μL of binding buffer was added prior to flow cytometric analysis. Three experimental replicates were performed per sample.

### 4.8. Cell Cycle Profiling

Using matching treatment conditions as apoptosis assays (10 μM of decursin vs. DMSO control), adherent cells were trypsinized after 24 h of exposure. Pelleted cells underwent two PBS washes and resuspension at 1 × 10^6^ cells/mL. Cell suspensions were stained with 1 mL of DNA staining solution and 10 μL of permeabilization reagent. After 10 s of vortex mixing and 30 min of dark incubation, cell cycle distributions were analyzed by flow cytometry with ModFit LT 5.0. Triplicate measurements ensured data reliability.

### 4.9. Western Blot

Western blot analysis was performed using RIPA buffer (containing protease inhibitors) for cell lysis, with total protein concentration quantified by BCA assay (Pierce™, Thermo Scientific, Waltham, MA, USA) using BSA standards and normalized against α-tubulin as a loading control. Proteins (25 μg/sample) were separated on 10% SDS-PAGE gels and transferred to PVDF membranes using a semi-dry transfer system. Membranes were blocked with Rapid Transfer Blocking Buffer (Thermo Scientific™) for 20 min at room temperature before overnight incubation at 4 °C with primary antibodies: TP63 (1:2000), SOX2 (1:1000–1:5000), and α-tubulin (1:3000–1:12,000) (all from Proteintech). After TBST washes, membranes were incubated with HRP-conjugated secondary antibodies (goat anti-mouse/rabbit IgG, 1:5000, Beyotime, Shanghai, China) for 1.5 h at room temperature. Protein bands were visualized using an ECL kit (Epizyme Biopharmaceutical, Shanghai, China) and imaged with a chemiluminescence detection system. Three independent biological replicates were conducted with experimental controls included in each run.

### 4.10. Statistical Analysis

All data were validated for normality using Shapiro–Wilk tests (*p* > 0.05). Two-group comparisons utilized unpaired/paired *t*-tests (validated by F-tests for homoscedasticity); multi-group data were analyzed via two-way ANOVA with Tukey’s post-hoc adjustment; dose–response relationships were modeled via four-parameter logistic regression to calculate IC50. Proportional data (e.g., cell cycle percentages) underwent arcsine-square-root transformation to stabilize variance (validated by Levene’s test, *p* > 0.1). Cellular parameters and Western blot band intensities were quantified through ImageJ version 1.40-based thresholding particle analysis and grayscale densitometry. Statistical significance was defined as *p* < 0.05 across three biological replicates. Analyses were performed using GraphPad Prism 9.0.

## 5. Conclusions

Decursin demonstrated potent antitumor activity against ESCC by inducing cell cycle deceleration, apoptosis, and ubiquitin–proteasome-mediated degradation of oncoproteins TP63 and SOX2. Its selective cytotoxicity toward cancer cells, combined with in vivo efficacy and minimal toxicity, positions it as a promising therapeutic candidate for ESCC. These findings support further preclinical and clinical exploration of decursin as a targeted monotherapy or adjunct in multimodal treatment regimens.

## Data Availability

Data is contained within the article and Supplementary Materials.

## Supplementary Materials

The following supporting information can be downloaded at: https://www.mdpi.com/article/10.3390/ijms26115391/s1.

## Abbreviations

The following abbreviations are used in this manuscript:

ESCC	Esophageal squamous cell carcinoma
EAC	Esophageal adenocarcinoma
HIF-1α	Hypoxia-inducible factor 1α
VEGF	Vascular endothelial growth factor
CA9	Carbonic anhydrase IX
ALT	Alanine aminotransferase
AST	Aspartate aminotransferase
Cr	Creatinine

## References

[1] Bray F., Laversanne M., Sung H., Ferlay J., Siegel R.L., Soerjomataram I., Jemal A. (2024). Global cancer statistics 2022: GLOBOCAN estimates of incidence and mortality worldwide for 36 cancers in 185 countries. CA Cancer J. Clin..

[2] Smyth E.C., Lagergren J., Fitzgerald R.C., Lordick F., Shah M.A., Lagergren P., Cunningham D. (2017). Oesophageal cancer. Nat. Rev. Dis. Primers.

[3] Wei H., Zhao D., Zhi Y., Wu Q., Ma J., Xu J., Liu T., Zhang J., Wang P., Hu Y. (2025). RTN4IP1 Contributes to ESCC via Regulation of Amino Acid Transporters. Adv. Sci..

[4] Sohda M., Kuwano H. (2017). Current Status and Future Prospects for Esophageal Cancer Treatment. Ann. Thorac. Cardiovasc. Surg..

[5] Yang Y.M., Hong P., Xu W.W., He Q.Y., Li B. (2020). Advances in targeted therapy for esophageal cancer. Signal Transduct. Target. Ther..

[6] Siegel R.L., Kratzer T.B., Giaquinto A.N., Sung H., Jemal A. (2025). Cancer statistics, 2025. CA Cancer J. Clin..

[7] Li M., Guo T., Lin J., Huang X., Ke Q., Wu Y., Fang C., Hu C. (2023). Corrigendum to “Curcumin inhibits the invasion and metastasis of triple negative breast cancer via Hedgehog/Gli1 signaling pathway” [J. Ethnopharmacol. 283 (2022) 114689]. J. Ethnopharmacol.

[8] Zou G., Zhang X., Wang L., Li X., Xie T., Zhao J., Yan J., Wang L., Ye H., Jiao S. (2020). Herb-sourced emodin inhibits angiogenesis of breast cancer by targeting VEGFA transcription. Theranostics.

[9] Shen Z., Zhao L., Yoo S.A., Lin Z., Zhang Y., Yang W., Piao J. (2024). Emodin induces ferroptosis in colorectal cancer through NCOA4-mediated ferritinophagy and NF-κb pathway inactivation. Apoptosis.

[10] Ji X., Chen Z., Lin W., Wu Q., Wu Y., Hong Y., Tong H., Wang C., Zhang Y. (2024). Esculin induces endoplasmic reticulum stress and drives apoptosis and ferroptosis in colorectal cancer via PERK regulating eIF2α/CHOP and Nrf2/HO-1 cascades. J. Ethnopharmacol..

[11] Chen P., Zhong X., Song Y., Zhong W., Wang S., Wang J., Huang P., Niu Y., Yang W., Ding Z. (2024). Triptolide induces apoptosis and cytoprotective autophagy by ROS accumulation via directly targeting peroxiredoxin 2 in gastric cancer cells. Cancer Lett..

[12] Muralikrishnan A., Sekar M., Kumarasamy V., Gan S.H., Ravi S., Subramaniyan V., Wong L.S., Wu Y.S., Khattulanuar F.S., Mat Rani N.N.I. (2024). Chemistry, Pharmacology and Therapeutic Potential of Decursin: A Promising Natural Lead for New Drug Discovery and Development. Drug Des. Devel Ther..

[13] Shehzad A., Parveen S., Qureshi M., Subhan F., Lee Y.S. (2018). Decursin and decursinol angelate: Molecular mechanism and therapeutic potential in inflammatory diseases. Inflamm. Res..

[14] Ge Y., Yoon S.H., Jang H., Jeong J.H., Lee Y.M. (2020). Decursin promotes HIF-1α proteasomal degradation and immune responses in hypoxic tumour microenvironment. Phytomedicine.

[15] Kim S., Kim J.E., Kim N., Joo M., Lee M.W., Jeon H.J., Ryu H., Song I.C., Song G.Y., Lee H.J. (2019). Decursin inhibits tumor growth, migration, and invasion in gastric cancer by down-regulating CXCR7 expression. Am. J. Cancer Res..

[16] Jiang Y.Y., Jiang Y., Li C.Q., Zhang Y., Dakle P., Kaur H., Deng J.W., Lin R.Y., Han L., Xie J.J. (2020). TP63, SOX2, and KLF5 Establish a Core Regulatory Circuitry That Controls Epigenetic and Transcription Patterns in Esophageal Squamous Cell Carcinoma Cell Lines. Gastroenterology.

[17] Liu Y., Xiong Z., Beasley A., D’Amico T., Chen X.L. (2016). Personalized and targeted therapy of esophageal squamous cell carcinoma: An update. Ann. N. Y. Acad. Sci..

[18] Abnet C.C., Arnold M., Wei W.Q. (2018). Epidemiology of Esophageal Squamous Cell Carcinoma. Gastroenterology.

[19] Chen W., Zheng R., Baade P.D., Zhang S., Zeng H., Bray F., Jemal A., Yu X.Q., He J. (2016). Cancer statistics in China, 2015. CA Cancer J. Clin..

[20] Barker H.E., Paget J.T., Khan A.A., Harrington K.J. (2015). The tumour microenvironment after radiotherapy: Mechanisms of resistance and recurrence. Nat. Rev. Cancer.

[21] Xia Y., Sun M., Huang H., Jin W.L. (2024). Drug repurposing for cancer therapy. Signal Transduct. Target. Ther..

[22] Wang Q., Du H., Geng G., Zhou H., Xu M., Cao H., Zhang B., Song G., Hu T. (2014). Matrine inhibits proliferation and induces apoptosis via BID-mediated mitochondrial pathway in esophageal cancer cells. Mol. Biol. Rep..

[23] Kang Q., He L., Zhang Y., Zhong Z., Tan W. (2024). Immune-inflammatory modulation by natural products derived from edible and medicinal herbs used in Chinese classical prescriptions. Phytomedicine.

[24] Atanasov A.G., Zotchev S.B., Dirsch V.M., Supuran C.T. (2021). Natural products in drug discovery: Advances and opportunities. Nat. Rev. Drug Discov..

[25] Kim S., Lee S.I., Kim N., Joo M., Lee K.H., Lee M.W., Jeon H.J., Ryu H., Kim J.M., Sul J.Y. (2021). Decursin inhibits cell growth and autophagic flux in gastric cancer via suppression of cathepsin C. Am. J. Cancer Res..

[26] Yang Y., Hu Y.E., Zhao M.Y., Jiang Y.F., Fu X., You F.M. (2023). Decursin affects proliferation, apoptosis, and migration of colorectal cancer cells through PI3K/Akt signaling pathway. Zhongguo Zhong Yao Za Zhi.

[27] Kim D., Go S.H., Song Y., Lee D.K., Park J.R. (2024). Decursin Induces G1 Cell Cycle Arrest and Apoptosis through Reactive Oxygen Species-Mediated Endoplasmic Reticulum Stress in Human Colorectal Cancer Cells in In Vitro and Xenograft Models. Int. J. Mol. Sci..

[28] Li W., Yang Y., Huang L., Yu X., Wang T., Zhang N., Yang M. (2024). The TDP-43/TP63 Positive Feedback Circuit Promotes Esophageal Squamous Cell Carcinoma Progression. Adv. Sci..

[29] Chen L., Zhu S., Liu T., Zhao X., Xiang T., Hu X., Wu C., Lin D. (2023). Aberrant epithelial cell interaction promotes esophageal squamous-cell carcinoma development and progression. Signal Transduct. Target. Ther..

[30] Wu Z., Zhou J., Zhang X., Zhang Z., Xie Y., Liu J.B., Ho Z.V., Panda A., Qiu X., Cejas P. (2021). Reprogramming of the esophageal squamous carcinoma epigenome by SOX2 promotes ADAR1 dependence. Nat. Genet..

[31] Choudhari A.S., Mandave P.C., Deshpande M., Ranjekar P., Prakash O. (2019). Phytochemicals in Cancer Treatment: From Preclinical Studies to Clinical Practice. Front. Pharmacol..

[32] Talib W.H., Awajan D., Hamed R.A., Azzam A.O., Mahmod A.I., Al-Yasari I.H. (2022). Combination Anticancer Therapies Using Selected Phytochemicals. Molecules.

[33] Castillo-Pichardo L., Dharmawardhane S.F. (2012). Grape polyphenols inhibit Akt/mammalian target of rapamycin signaling and potentiate the effects of gefitinib in breast cancer. Nutr. Cancer.

[34] La X., Zhang L., Li Z., Li H., Yang Y. (2019). (−)-Epigallocatechin Gallate (EGCG) Enhances the Sensitivity of Colorectal Cancer Cells to 5-FU by Inhibiting GRP78/NF-κB/miR-155-5p/MDR1 Pathway. J. Agric. Food Chem..

[35] Xu W., Xie S., Chen X., Pan S., Qian H., Zhu X. (2021). Effects of Quercetin on the Efficacy of Various Chemotherapeutic Drugs in Cervical Cancer Cells. Drug Des. Devel Ther..

